# 

**Published:** 1891-12

**Authors:** 


### TABLE OF CONTENTS.

ORIGINAL COMMUNICATIONS. PAGE

The Chemical Metamorphosis of the Fluids of the Mouth with Relation to Discoloration of Gold. By EDWARD S. NILES, D.M.D. . . . . . . . . . 805

How shall we practise Dentistry in order to make it Healthful? By DR. CHARLES T. TERRY, Milan, Italy . . . . . . . . . . . . . . . . . . . 809

Abrasions of the Teeth. By DR. A. F. TOWNSEND, Worcester, Mass. . . . 811

Rapid Operations. By FRANK PERRIN, D.M.D. . . . . . . . . . . . . 815

REPORTS OF SOCIETY MEETINGS.

American Dental Association . . . . . . . . . . . . . . . . . . . . . . 818

EDITORIAL.

Where should Papers be published? . . . . . . . . . . . . . . . . . . 847

The Closing of the Year . . . . . . . . . . . . . . . . . . . . . . 850

Assistant Editor . . . . . . . . . . . . . . . . . . . . . . . . . . 851

BIBLIOGRAPHY . . . . . . . . . . . . . . . . . . . . . . . . . . . . 852

OBITUARY.

American Dental Association—In Memoriam: Dr. W. H. Atkinson . . . 855

DOMESTIC CORRESPONDENCE.

American Medical Association . . . . . . . . . . . . . . . . . . . . 857

On "Inlays" . . . . . . . . . . . . . . . . . . . . . . . . . . . . . 859

A Correction . . . . . . . . . . . . . . . . . . . . . . . . . . . . 860

CURRENT NEWS.

Missouri State Dental Association . . . . . . . . . . . . . . . . . . 861

Massachusetts Dental Society . . . . . . . . . . . . . . . . . . . . 861

Mississippi Valley Medical Association . . . . . . . . . . . . . . . . 862

Invitation to Anniversary Meeting of the First District Society of New York . . . . . . . . . . . . . . . . . . . . . . . . . . . . . . . 862

Items . . . . . . . . . . . . . . . . . . . . . . . . . . . . . . . 863

